# MDM4 actively restrains cytoplasmic mTORC1 by sensing nutrient availability

**DOI:** 10.1186/s12943-017-0626-7

**Published:** 2017-03-07

**Authors:** Francesca Mancini, Emanuela Teveroni, Giusy Di Conza, Valentina Monteleone, Ivan Arisi, Marsha Pellegrino, Marianna Buttarelli, Luisa Pieroni, Mara D’Onofrio, Andrea Urbani, Alfredo Pontecorvi, Massimiliano Mazzone, Fabiola Moretti

**Affiliations:** 1 Institute of Cell Biology and Neurobiology, National Research Council of Italy (CNR), 00143 Rome, Italy; 2 PostGraduate School of Endocrinology and Metabolic Diseases, Institute of Pathology, Catholic University of Rome, 00168 Rome, Italy; 3 Laboratory of Molecular Oncology and Angiogenesis, Department of Oncology, KU Leuven, 3000 Leuven, Belgium; 4 Laboratory of Molecular Oncology and Angiogenesis, Vesalius Research Center, VIB, 3000 Leuven, Belgium; 5 European Brain Research Institute (EBRI) Rita Levi-Montalcini, 00143 Rome, Italy; 6 Proteomic and Metabonomic Laboratory, Fondazione Santa Lucia, 00143 Rome, Italy; 7 Institute of Biochemistry and Biochemical Clinic, Catholic University of Rome, 00168 Rome, Italy

**Keywords:** MDM4, mTOR, Nutrient deprivation, Aminoacid, p53

## Abstract

**Background:**

Many tumor-related factors have shown the ability to affect metabolic pathways by paving the way for cancer-specific metabolic features. Here, we investigate the regulation of mTORC1 by MDM4, a p53-inhibitor with oncogenic or anti-survival activities depending on cell growth conditions.

**Method:**

MDM4-mTOR relationship was analysed through experiments of overexpression or silencing of endogenous proteins in cell culture and using purified proteins in vitro. Data were further confirmed in vivo using a transgenic mouse model overexpressing MDM4. Additionally, the Cancer Genome Atlas (TCGA) database (*N* = 356) was adopted to analyze the correlation between MDM4 and mTOR levels and 3D cultures were used to analyse the p53-independent activity of MDM4.

**Results:**

Following nutrient deprivation, MDM4 impairs mTORC1 activity by binding and inhibiting the kinase mTOR, and contributing to maintain the cytosolic inactive pool of mTORC1. This function is independent of p53. Inhibition of mTORC1 by MDM4 results in reduced phosphorylation of the mTOR downstream target p70S6K1 both in vitro and in vivo in a MDM4-transgenic mouse. Consistently, MDM4 reduces cell size and proliferation, two features controlled by p70S6K1, and, importantly, inhibits mTORC1-mediated mammosphere formation. Noteworthy, MDM4 transcript levels are significantly reduced in breast tumors characterized by high mTOR levels.

**Conclusion:**

Overall, these data identify MDM4 as a nutrient-sensor able to inhibit mTORC1 and highlight its metabolism-related tumor-suppressing function.

**Electronic supplementary material:**

The online version of this article (doi:10.1186/s12943-017-0626-7) contains supplementary material, which is available to authorized users.

## Background

In the last years, many studies have reported the crosstalk between the pathways that control tumor development and cellular metabolism. MDM4 (also MDMX) is a crucial regulator of the tumor suppressor Tp53 [1, 2]. It cooperates with MDM2 by forming a MDM2/MDM4 heterodimer that efficiently reduces p53 levels and activity [3, 4]. As such, it possesses oncogenic features and accordingly its cancer promoting function has been reported [5, 6]. Conversely, under severe DNA damage, the two MDM proteins dissociate and MDM4 promotes p53-proapoptotic function by favouring the pro-apoptotic phosphorylation of p53 by the kinase HIPK2 [7] and the mitochondrial activity of p53 [8, 9]. According to these last activities, its presence is correlated to the beneficial effects of chemotherapy in wild type p53 tumors [8–10]. Under mild cytostatic DNA damage, the protein is actively degraded and this allows p53 to execute its growth arrest response [2]. Finally, MDM4 promotes chromosome and genome stability in long-term in vitro cultures, and suppresses tumorigenesis, independently of p53 [11]. Thus, MDM4 appears to be sensitive to the cell growth conditions and its function to be consequently determined. To date, no direct activity has been reported for p53 and its MDM regulators towards mTORC1 function.

The kinase target of rapamycin (TOR) is one of the hubs that control cell physiology based on availability of nutrients, growth factors, and energy [12]. Mammalian (recently, also mechanistic) TOR, mTOR, develops its kinase activity within two hetero complexes: mTORC1 and mTORC2 with mTORC1 integrating the signals from all previous factors. Mammalian TORC1 promotes cell growth and proliferation, a reason whereby its activity and/or levels are frequently increased in human tumors [13]. Two main targets of mTORC1 are p70S6 kinase 1 (also S6K1) and eukaryotic initiation factor 4E-binding protein 1 (eIF4), both regulating mRNA translation initiation and progression, thus the rate of protein synthesis [12, 14]. The active form of mTORC1 resides at the lysosomes where it directly prevents autophagy and controls lysosome function [15]. In response to nutrient deprivation, mTORC1 is released from activating partners and re-localizes from the lysosomal surface to the cytosolic compartment. The features underlying mTORC1 cytoplasmic localization are presently undefined.

Starting from a shotgun proteomic comparative analysis of the untransformed breast cell line MCF10A, we have demonstrated that knocking down of MDM4 alters the function of the p70S6K signalling. Our results demonstrate that MDM4 contributes to maintain mTORC1 in its inactive state in the cytoplasm, thus providing MDM4 of the ability to sense metabolic stress and to control mTORC1-dependent oncogenic properties.

## Methods

### Cell cultures, transfections and treatments

HeLa, 293 T, HCT116, MDA-MB231 cells were maintained in DMEM/10% FBS (Life Technologies, USA), p53^−/−^Mdm4^−/−^MEFs, p53^−/−^Mdm2^−/−^MEFs, and p53^−/−^MEFs in DMEM high glucose/10% FBS (Cambrex). MCF10A cells in MEGM (Lonza, Switzerland). MDM4 and control (CTL) siRNA were by Invitrogen (Stealth RNAi), siRNA for S6K1 were from Ambion. siRNA and plasmids transfection were performed with RNAiMAX and Lipofectamine Plus respectively according to manufacturer’s instructions (Invitrogen). mTOR_1 shRNA was obtained from D. Sabatini through Addgene. Rapamycin (Sigma) was used 50nM unless specifically indicated. Torin2 was used 50nM. For amino acid starvation, cells were incubated for 3 h in amino acid free RPMI (US Biological) supplemented with 10% inactivated FBS, and stimulated with amino acid mixture for the indicated time. For serum and amino acid starvation, cells were incubated in EBSS (Invitrogen) for 50’, and stimulated with amino acids mixture or complete medium for the indicated time.

### Shotgun proteomic analysis

MCF10A cells were transfected with stealth MDM4-specific (si*MDM4*-MCF10A) or stealth control RNA (si*CTL*-MCF10A), and after 48 h were lysed. The proteomic analysis was performed on proteins extracted from cytoplasmic cell lysate of MCF10 cells, through a label-free data-independent differential proteomic analysis by nUPLC-MS^E^. Details of the analysis are reported in [7].

### Mammosphere forming assay

For mammosphere formation assay, cell culture dishes have been coated with pHEMA (poly(2-hydroxyethyl methacrylate) 10 mg/ml, dried and rinse with PBS. MDA-MB231 were interfered for siRNA control or siMDM4 for 16 h. Afterwards, cells were detached and seeded at 2000 cells/well in pHEMA coated 6 wells dishes for 72 h in DMEM/F12 supplemented with 2 mM Glutamine, 100U/ml Penicillin/streptomicin, 5%FBS, 20 ng/ml EGF, 0.5 mg/ml Hydrocortisone, 10ug/ml Insulin.

### Immunoprecipitation, western blot and cell fractionation

For immunoprecipitation (IP), cells were lysed in CHAPS lysis buffer (40 mM Hepes pH7.4, 120 mM NaCl, 2 mM EDTA, 0.3% CHAPS) containing mix of protease inhibitors (Boehringer), plus 5 mM NaF, 10 mM glycerophosphate and 1 mM Na_3_VO_4_. For IP lysates were pre-incubated with protein G-Agarose (Pierce) and then with the indicated antibody, under gentle rocking at 4 °C overnight. For Western blot (Wb) cells were lysed in RIPA buffer. Membranes were developed using the enhanced chemiluminescence (ECL Amersham) by chemiluminescence imaging system, Alliance 2.7 (UVITEC Cambridge) and quantified by the software Alliance V_1607. Primary antibodies used: MDM4 BL1258 (Bethyl laboratory), MDM4 C82 (Sigma), MDM4 8C6 (Millipore) p53 FL393 (Santa Cruz), α-tubulin DM1A (Sigma), actin C-40 (Sigma), mTOR (Santa Cruz), mTOR (Cell Signaling), anti-FLAG M2 affinity gel (Sigma), phosphoSer473-AKT (Cell Signaling), phosphor-Thr389-S6K (Cell Signaling), Akt (Cell Signaling), S6K1 (Santa Cruz), Raptor (Cell Signaling), Raptor (Santa Cruz).

Fractionation of lysates into heavy membrane and light membrane/cytosolic fractions was performed according to Menon et al. 2014.

### In vitro kinase assay

Kinase assays were performed as previously described [16] with some modifications. Flag-mTOR immunoprecipitate was washed twice in CHAPS lysis buffer and twice in 25 mM HEPES (pH 7.4), 20 mM potassium chloride. Kinase assays were performed at 30 °C for 20 min in a final volume of 30 μl consisting of mTORC1 kinase buffer (25 mM HEPES [pH 7.4], 50 mM KCl, 10 mM MgCl_2_, 250 μM ATP) and inactive GST-S6K1 purified (by GST-Agarose gel, Sigma), from Hela cells transfected with GST-S6K1 plasmid and treated with EBSS and 20 μM LY294002 for 1 h. Reactions were stopped by the addition of sample buffer and boiling for 5 min. When used, 150 ng of GST-MDM4 was added to mTORC1 10 min before the addition of ATP to the kinase assay.

### Cell viability and cell cycle analysis

Cell proliferation was determined by Cell Titer Blue colorimetric assay or Cell Live/Dead kit according to the manufacturer’s instructions (Promega and Invitrogen, respectively). Cell cycle profiles and forward scatter determination (FSC-H) were evaluated by fixing cells in cold 70% ethanol for 1 h on ice and staining DNA for 30 min at room temperature with 50 μg/mL propidium iodide (PI) in PBS containing 1 mg/mL RNase A. FSC-H evaluation was performed by previous gating of cells in G1 phase. FACScan flowcytometer (Becton Dickinson, USA) was used and data analysed by CellQuest Software (Becton-Dickinson).

### Immunofluorescence

Hela cells were fixed with 4% formaldehyde 5’ 37 °C, permeabilized with TritonX100 0.2% 15’ RT, and blocked with 0.25% BSA. Cells were stained with DAPI and primary antibodies: anti-MDM4 (1:100 Origene 4B5), and anti-mTOR (1:400 Cell Signaling). Cyanine (Cy3)-conjugated and Cyanine (Cy2)-conjugated secondary antibodies were used.

### Mouse maintenance and treatment

Control (WT) and Mdm4 transgenic (TG) mice [5] were maintained and treated in accordance with the Guidelines on the protection of animals used for scientific purposes (European Directive 63/2010/EU and Italian Law DL116/1992 and DL 26/2014). Relative ethical approval has been obtained by Animal Welfare Body “Fondazione S. Lucia” (Protocol Number: 969/2015-PR). For in vivo assessment of mTOR activity, 13–15 week old male mice were fasted overnight and after 16 h intraperitoneally injected with leucine (120 mg/kg) or saline solution (control) in 0.2 ml volume. Ten minutes after injection, mice were sacrificed, tissues were snap frozen in liquid nitrogen and samples processed in RIPA lysis buffer for Western blot analysis.

### Lentivirus infection

The FH1t-UTG Mdm4 3' UTR-GFP lentiviral construct was obtained by Marine’s Lab by cloning shRNA sequence for MDM4-3'UTR (ACAGTCCTTCAGCTATTTCATTTCAAGAGAATGAAATAGCTGAAGGACTGTTTTTT) into the FH1tUTG vector, which constitutively expresses GFP [17]. MCF10A, Hela and 293 T cells were infected with FH1t-UTG Mdm4 3' UTR-GFP lentivirus to generate TET-shMDM4 inducible cell line by doxycycline (DOX).

## Results

### MDM4 inhibits p70S6K1 phosphorylation

Survey by Ingenuity Pathways Analysis™ of proteomic profile of immortalized MCF10A breast cell line interfered by siRNA to MDM4 compared to control cells (siMDM4 vs siCTL) revealed that some proteins upregulated by knockdown of MDM4 belong to the function “Regulation of eIF4 and p70S6K Signalling” (Table 1) [7, 18–20].

**Table 1:** Regulation of eIF4 and p70S6K signalling pathway: protein targets

Symbol	Entrez Gene Name	siMDM4/ siCTL Fold of induction	Location	Type(s)
ITGA5	integrin, alpha 5	2.4	Plasma Membrane	transmembrane receptor
RPS12	ribosomal protein S12	10.0	Cytoplasm	other
RPSA	ribosomal protein SA	10.0	Cytoplasm	translation regulator

**Fig 1:**
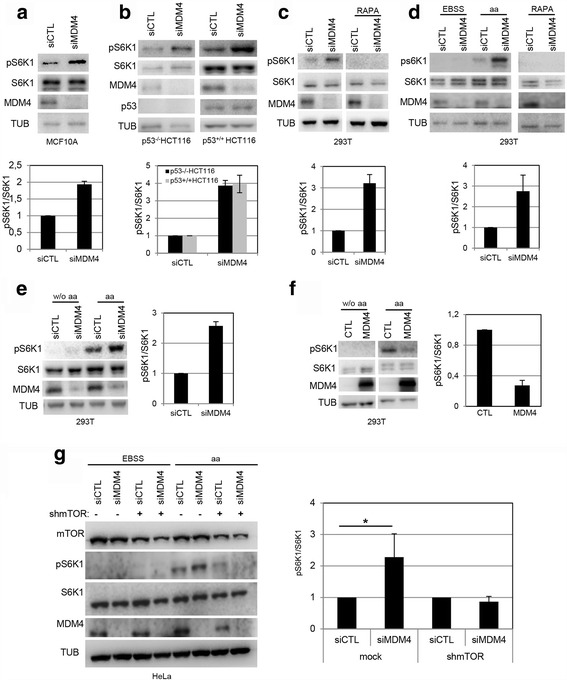
MDM4 inhibits S6K1 phosphorylation. **a** Representative Wb analysis of the indicated proteins in MCF10A cells transfected with siCTL or siMDM4 and collected after 48 h (hrs). Histogram in the *lower panel* shows the ratio of densitometric values of phosphorylated S6K1 (pS6K1) to S6K1. The ratio pS6K1/S6K1 from siCTL *lane* was arbitrarily set to 1. Mean ± SD of two independent biological replicates is shown (*N* = 2). **b** Wb analysis of the indicated proteins in p53^−/−^HCT116 and p53^+/+^HCT116 cells transfected as in (a). Histogram as in (a) (*N* = 2). **c** Wb analysis of the indicated proteins in 293 T cells transfected with siMDM4 or siCTL and after 24 h treated with Rapamycin (RAPA, 40nM) for additional 24 h. Histogram as in (a) (*N* = 2). **d** Wb analysis of the indicated proteins in 293 T cells transfected as in (c) and after 48 h treated with EBSS for 50’ and then in presence or absence of amino acids mixture (aa) for 30’, or with RAPA for 1 h. Histogram as in (a) (*N* = 2). **e** Wb analysis of the indicated proteins in 293 T cells transfected as in (**c**). After 48 h, cells were grown in medium deprived of amino acids (w/o aa) for 3 h, and in absence or presence of aa for the last 10’. Histogram as in (a) (*N* = 2). **f** Wb analysis of the indicated proteins in 293 T cells transfected with pcDNA3.1-MDM4 (MDM4) or control vector (CTL) and after 24 h treated as in (**e**) (*the two panels derive from the same blot*). Histogram as in (a). The ratio pS6K1/S6K1 from CTL lane was arbitrarily set to 1 (*N* = 2). **g** Wb analysis of the indicated proteins in Hela cells transfected with siMDM4 or siCTL and with shcontrol vector (Mock) or shmTOR for 48 h, then treated with EBSS for 50’, and for additional 30’ with aa. Histogram as in (a). (*N* = 3, * = *p* < 0.05, two‐tailed unpaired t‐test)

To validate these proteomic data, we evaluated the levels of the 70 kDa ribosomal protein S6 kinase, p70S6K1 (hereafter, S6K1) following knockdown (KD) of MDM4 in MCF10A. Since phosphorylation of S6K1 at the threonine 389 is a key signal in the activation of the S6K1 downstream signalling pathway [21], we analysed the levels of both S6K1 and its active form, pS6K1^389^. In fact, MDM4-KD caused up-regulation of pS6K1 while did not alter the total levels of the protein (Fig. 1a), confirming bioinformatics prediction analysis. Similar results were obtained by constitutive inducible knockdown of MDM4 in MCF10A (Additional file 1: Fig. S1a) and in HeLa cells (Additional file 1: Fig. S1b) that express doxycycline-inducible shRNA targeting a different region of MDM4, thus excluding off target effect of siRNAs. Furthermore, re-expression of MDM4 rescued the increase of pS6K1 levels caused by MDM4-KD (Additional file 1: Fig. S1b), confirming the specific activity of MDM4 on S6K1 phosphorylation.

These data were observed also in p53^+/+^HCT116 and syngenic p53^−/−^HCT116 cells, pointing to a p53-independent effect of MDM4 on S6K1 (Fig. 1b).

Since S6K1 phosphorylation is mostly controlled by the mammalian target of rapamycin complex 1, mTORC1 [22], we analysed whether MDM4 activity is developed through inhibition of this complex. Human embryonic kidney 293 T cells, a mammalian cell line in which mTORC1 activity has been well characterized [23], were transfected with MDM4 siRNA and the levels of pS6K1 analysed in the presence of the mTORC1 inhibitor rapamycin (RAPA). In this cell line too, MDM4-KD increased the levels of pS6K1 but it was ineffective in the presence of RAPA (Fig. 1c). Basal S6K1 phosphorylation was inhibited by RAPA confirming the block of mTORC1 function. Similar results were obtained in MCF10A (Additional file 1: Fig. S1a) and in HeLa cells (Additional file 1: Fig. S1c). These data suggest that MDM4 inhibits mTORC1-mediated S6K1 phosphorylation. Given the inactivation of p53 both in 293 T and in HeLa cells, these data further support the p53-independent activity of MDM4.

The mTORC1-mediated phosphorylation of S6K1 is tightly regulated by nutrient availability and has been particularly well characterized by amino acids signalling [24, 25]. To further analyse the inhibitory function of MDM4 towards mTORC1, the activity of this last was blocked by cell starvation and then re-stimulated by amino acids (aa) addition. Indeed, cell treatment with Earle's Balanced Salt Solution (EBSS) depleted pS6K1 levels that were rescued by addition of amino acids (aa) mixture (Fig. 1d, Additional file 1: Fig. S1d). Under these conditions, MDM4-KD enhanced the increase of pS6K1 caused by aa addition, indicating that MDM4 antagonizes S6K1 phosphorylation by restraining mTORC1 activity (Fig. 1d, Additional file 1: Fig. S1d). Consistently, MDM4-KD was ineffective in the presence of RAPA (Fig. 1d). Similarly, amino acid deprivation restrained mTORC1 activity and the presence of MDM4 reduced the recovery of pS6K1 (Fig. 1e). Conversely, the over-expression of MDM4 strongly decreased the levels of pS6K1 induced by aa supplementation (Fig. 1f), overall indicating that MDM4 inhibits mTORC1 in response to aa depletion. To further confirm that MDM4 effect on pS6K1 are mediated through regulation of mTOR, the knockdown of mTOR prevented the upregulation of pS6K1 by siMDM4 (Fig. 1g). Similar effect were observed by pharmacological inhibition of mTOR with Torin2, a potent ATP-competitive inhibitor [26] although with less efficiency (Additional file 1: Fig. S1e).

**Fig 2:**
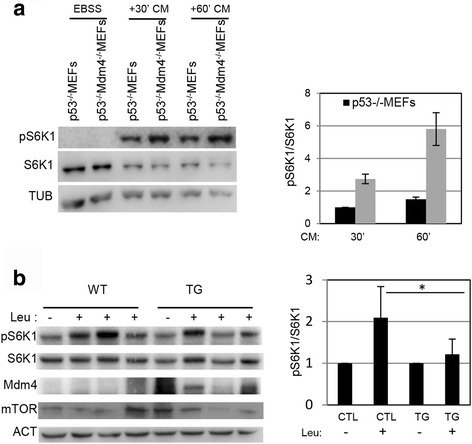
**a** Wb analysis of the indicated proteins in *p53* ^*−/−*^ MEFs and *p53* ^*−/−*^ *Mdm4* ^*−/−*^MEFs treated with EBSS for 50’ and then with complete growth medium (CM) for the indicated time points. Histogram reports the ratio of densitometric values of phosphorylated S6K1 (pS6K1) to S6K1. The ratio pS6K1/S6K1 from *p53* ^*−/−*^ MEFs *lane* at 30’ was arbitrarily set to 1. Mean ± SD of two independent biological replicates is shown (*N* = 2). **b** Wb analysis of the indicated proteins in the liver from four control mice (WT) and four MDM4 transgenic mice (TG) treated with saline solution (−) or Leucine (+) (Leu, 120 mg/kg). Histogram as in (a) The ratio pS6K1/S6K1 from wt (CTL) untreated sample was arbitrarly set to 1﻿. Mean ± SD is shown (*N* = 4) (* = *p* < 0.05, two‐tailed unpaired t‐test)

To further confirm this MDM4 activity in normal cells, we used the genetic model of *Mdm4* knock out in mouse embryo fibroblasts (MEFs) [27]. To exclude the effect of p53, *p53* ^*−/−*^ *Mdm4* ^*−/−*^MEFs were compared to *p53* ^*−/−*^MEFs. After cell treatment with EBSS, induction of pS6K1 by growth medium reconstitution was indeed significantly increased in *p53* ^*−/−*^ *Mdm4* ^*−/−*^MEFs compared to *p53* ^*−/−*^MEFs (Fig. 2a). In comparison, the *p53* ^*−/−*^ *Mdm2* ^*−/−*^MEFs showed pS6K1 levels similar to those of *p53* ^*−/−*^MEFs or even lower (Additional file 1: Fig. S1f), indicating the specificity of the activity of Mdm4 and excluding a general function of Mdm family. Overall, these data demonstrate that MDM4 represses the phosphorylation of S6K1 via inhibition of mTORC1 complex.

To ascertain that MDM4 displays this activity in vivo too, we used a transgenic mouse model overexpressing Mdm4 (TG) [5]. Since mTORC1 activity is strongly regulated in hepatocytes, we analysed pS6K1 levels in the liver of TG and age-matched control (WT) mice. Animals were fasted overnight and after 16 h injected intraperitoneally with the amino acid leucine (Leu), a specific activator of mTORC1 [28]. In control WT mice, Leu increased phosphorylation of S6K1 compared to saline treated mice (Fig. 2b). Of note, such increase was almost abolished in Mdm4 TG mice (Fig. 2b), indicating that the overexpression of Mdm4 restrains mTORC1 activity in vivo too. Evaluation of mTOR levels in these samples did not show significant differences between WT and TG mice thus excluding an impact of Mdm4 on the total amount of the kinase.

### MDM4 binds and inhibits mTOR

**Fig 3:**
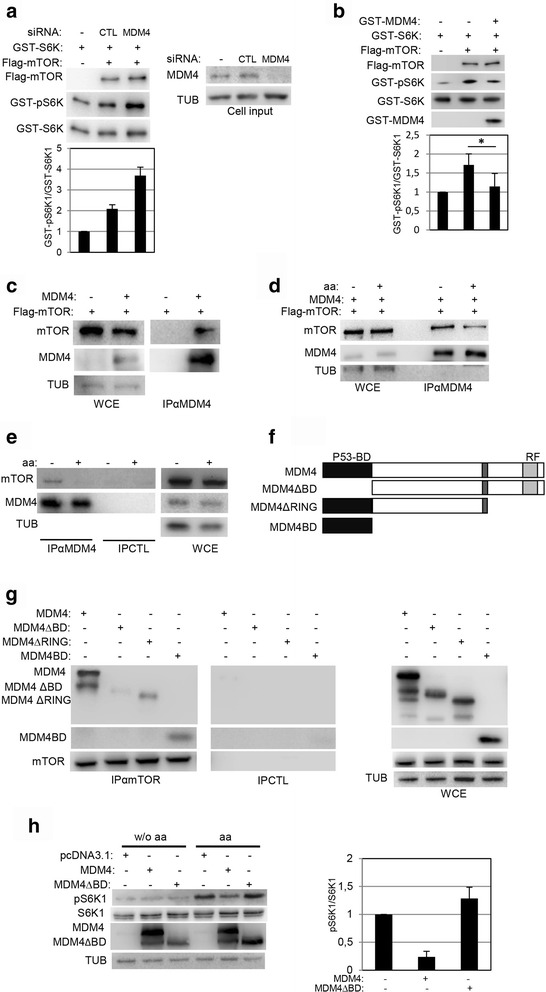
MDM4 binds and inhibits mTOR kinase activity. **a** Wb analysis of indicated proteins used for in vitro kinase assay (*left panel*). Flag-mTOR and GST-S6K were overexpressed an purified from HeLa cells. *Right panel* shows the levels of MDM4 in the cell input used for the in vitro kinase assay. Histogram in the *lower panel* shows the ratio of densitometric values of pS6K1 to S6K1. The ratio of pS6K1/S6K1 from siCTL lane was arbitrarily set to 1. Mean ± SD of two independent biological replicates is shown. **b** Wb analysis of the indicated proteins from in vitro kinase assay. Immunoprecipitated Flag-mTOR and GST-p70S6K1 (GST-S6K) were incubated with GST-MDM4 (*purified from bacteria*) for 10’ before the kinase assay. Histogram reports data as in (a) (*N* = 4) **c** Wb of indicated protein in co-immunocomplexes from HeLa cells transfected with the indicated plasmids. 500 μg of whole cell extract (WCE) was immunoprecipitated with anti-MDM4 antibody C82 (IPαMDM4, *left panel*). *Right panel* shows analysis of 1/10 of WCE. **d** Wb of indicated protein in co-immunocomplexes from HeLa cells transfected as in (c) and after 24 h grown in EBSS (−) for 1 h and then in absence or presence of the aa for additional 15’. **e** Wb of indicated protein in co-immunocomplexes from HeLa cells grown in EBSS for 50’ (−) and then in absence or presence of the aa for additional 15’. 1 mg of WCE was immunoprecipitated with anti-MDM4 antibody C82 (IPαMDM4) or Ig control (IPCTL). *Right panel* shows analysis of 1/15 of WCE. **f** Scheme of MDM4 deletion mutants. P53-BD means p53-binding domain, RF Ring Finger domain. **g** Wb of indicated protein in co-immunocomplexes from 293 T cells transfected with the indicated plasmids. 500 μg of WCE were immunoprecipitated with anti-mTOR antibody (IPαmTOR) or control Ig (IPCTL). *Right panel* shows the analysis of 1/10 of WCE. **h** Wb analysis of the indicated proteins in 293 T cells transfected with the indicated plasmids. After 48 h cells were grown in the medium without aa for 3 h and then in absence (w/o aa) or presence of aa for the last 15’. Histogram as in (a) (*N* = 2)

Previous data indicate that MDM4 inhibits mTORC1 activity by impairing its ability to phosphorylate the substrate S6K1. To understand whether MDM4 inhibits directly mTOR, we analysed the in vitro kinase function of the complex in presence or absence of MDM4. Flag-mTOR was immunopurified from HeLa cells overexpressing Flag-mTOR and tested in vitro for the phosphorylation of GST-p70S6K1 purified from HeLa cells. Incubation of GST-p70S6K1 with Flag-mTOR increased S6K1 phosphorylation compared to control cells (Fig. 3a). Interestingly, when mTORC1 complex was immunopurified from HeLa cells silenced for MDM4, the levels of phosphorylated GST-p70S6K1 were increased, suggesting that MDM4 directly impairs mTORC1 kinase activity (Fig. 3a). Consistently, pre-incubation of the mTORC1 complex with GST-MDM4 decreased significantly phosphorylation of S6K1 (Fig. 3b), indicating that the presence of MDM4 is sufficient to inhibit mTOR kinase activity.

Since both proteins are mainly cytoplasmic [15, 29, 30], these data prompted us to investigate a possible interaction between MDM4 and mTOR, the kinase effector of the mTORC1 complex. Indeed, overexpressed MDM4 co-immunoprecipitated Flag-mTOR, indicating that the two proteins interact (Fig. 3c). Interestingly, the amount of co-immunoprecipitated mTOR was lower in presence of aa, supporting the inhibitory activity of MDM4 towards mTORC1 under nutrient deprivation (Fig. 3d). Analysis of endogenous proteins confirmed the interaction between MDM4 and mTOR during starvation whereas this was almost undetectable in presence of aa (Fig. 3e). To ascertain whether the binding between the two proteins mediates the MDM4 inhibitory activity, map of the binding of MDM4 to mTOR was performed by using different MDM4 deletion mutants (Fig. 3f) whose cytoplasmic localization was previously reported [29]. The results revealed that the MDM4ΔBD, lacking the aminoacids 1–106 (consisting of the p53 binding domain) was unable to bind mTOR (Fig. 3f and g), indicating that the N-terminal domain of MDM4 is involved in the interaction. Of note, the MDM4ΔBD mutant did not decrease pS6K1 levels compared to the full-length MDM4 (Fig. 3h), indicating that the interaction between MDM4 and mTOR is required for the inhibition of this last. Overall, these data indicate that MDM4 binds mTOR during aa starvation and contributes to silence the kinase activity of the complex.

**Fig 4:**
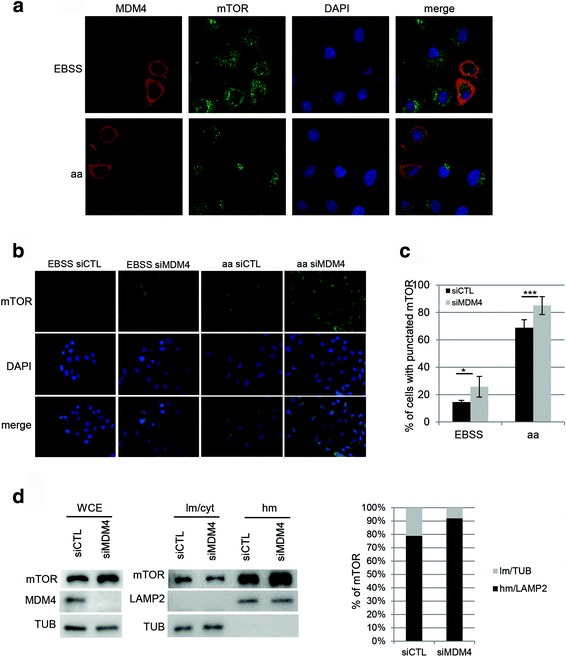
MDM4 interacts with cytoplasmic mTOR. **a** Representative pictures of immunofluorescence of HeLa cells transiently transfected with MDM4 and after 24 h treated with EBSS for 50’ and with aa for the last 15’. Endogenous mTOR is stained in *green*, MDM4 in *red*. DNA in blue (by DAPI). *Merge* shows the overlap of the signals. **b** Representative pictures of immunofluorescence of HeLa cells transfected with siCTL or siMDM4 and after 48 h treated with EBSS for 50’ and with aa for the last 15’. Endogenous mTOR is stained in *green*, DNA in *blue* (by DAPI). *Merge* shows the overlap of the signals. **c** Percentage of cells showing mTOR punctuated staining. Mean ± SD of three independent biological replicates is shown (** = *p* < 0.01; *** = *p* < 0.001 two‐tailed unpaired t‐test). **d** Wb analysis of the indicated proteins in HeLa cells transfected with siMDM4 or siCTL. After 48 h, cell lysates were fractionated in light membrane/cytosol (lm/cyt) and heavy membrane (hm) fractions. *Left panel* shows WCE. Histogram shows the percentage (%) of mTOR signal in the lm (*light bar*) and hm (*black bar*) fractions corrected for the respective loading control. mTOR signal in the lm + hm fractions was arbitrarily set to 100%

Depletion of amino acids induces re-localization of mTORC1 from lysosomal membranes to the cytosolic compartment and this correlates with decreased mTORC1 activity. MDM4 is mainly a cytoplasmic protein [29]. We therefore analysed in which compartment the interaction between MDM4 and mTOR occurs. Immunofluorescence showed that under starvation, overexpressed MDM4 and mTOR signals localize in the cytoplasm, whereas their signals are mostly independent when amino acids are not limiting in the culture medium (Fig. 4a). Particularly, upon amino acid supplementation mTOR assumed the characteristic punctate pattern, i.e. the lysosomal-active state [31], while MDM4 signal was not altered. Overall, these data suggest that MDM4 interacts with the soluble cytoplasmic pool of mTOR, contributing to keep it inactive. Accordingly, silencing of MDM4 significantly increased the percentage of cells with punctuated mTOR (Fig. 4b and c). This occurs both in EBSS conditions where a low fraction of mTOR is present at the lysosomes, as well as under aa treatment. Furthermore, fractionation of HeLa cell lysate [32] showed increased levels of mTOR in the lysosome-enriched fraction (hm, the heavy membrane) in siMDM4 compared to control cells (siCTL) and concomitantly reduced levels in the light membrane/cytosolic fraction (lm/cyt) (Fig. 4d). Similar results were obtained in 293 T cells (Additional file 1: Fig. S2). These data confirm the role of MDM4 as mTOR-cytoplasmic anchor and indicate that the balance between MDM4 and mTOR levels is important to determine the fraction of lysosomal active mTORC1.

### MDM4 affects cell size and proliferation

**Fig 5:**
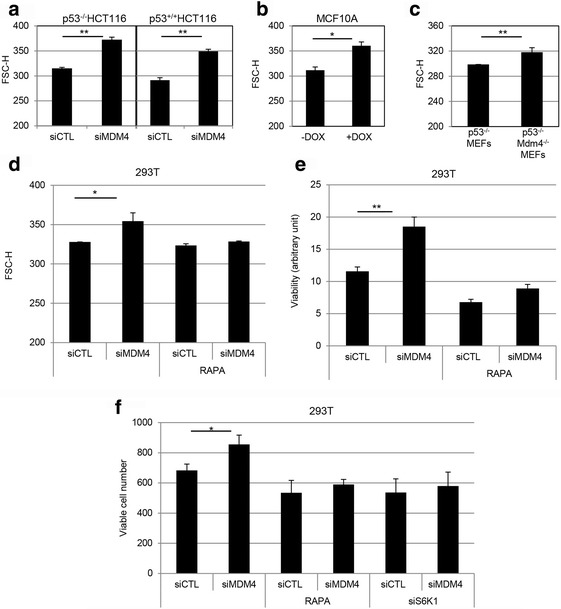
MDM4 regulates cell size and cell proliferation in a mTOR-dependent manner. **a, b** Forward scatter analysis (FSC-H) of p53^−/−^HCT116, p53^+/+^HCT116 (**a**) and MCF10A cells (**b**) transfected with siMDM4 or siCTL and after for 48 h analysed by flow cytometry. Mean ± SD of three independent biological replicates is shown (*N* = 3). **c** FSC-H of *p53* ^*−/−*^MEFs and *p53* ^*−/−*^ *Mdm4* ^*−/−*^MEFs (*N* = 3). **d** FSC-H of 293 T cells transfected with siMDM4 or siCTL and after 24 h treated with RAPA (40nM) for additional 24 h (*N* = 3). **e** Cell viability by Cell Titer *Blue* colorimetric assay of 293 T cells treated as in (**d**) (*N* = 3). **f** Evaluation of viable cells by Trypan *blue* in 293 T cells transfected with siMDM4 or siCTL and after 24 h treated with RAPA or transfected with siS6K1 for additional 24 h (*N* = 3, * = *p* < 0.05, ** = *p* < 0.01, two‐tailed unpaired t‐test)

The main functions of p70S6K1 are the stimulation of protein synthesis and the control of cell size and growth. To evaluate the impact of MDM4 towards these cell features, we analysed cell size by flow-cytometer. The results showed an increase of the mean cell size (FSC-H) in siMDM4 compared to siCTL p53^+/+^HCT116 cells (Fig. 5a) whereas the coefficient of variation of the FSC-H distribution was very similar in the two populations (CV_siMDM4_, 19,13 vs CV_siCTL_ 21,45). Similar results were observed in p53^−/−^HCT116 (Fig. 5a), thus excluding that these effects are due to MDM4 activity towards its main target p53. These data were further confirmed in DOX-inducible MDM4-KD MCF10A (Fig. 5b), in HeLa cells (Additional file 1: Fig. S3a), and in *p53* ^*−/−*^ *Mdm4* ^*−/−*^MEFs compared to *p53* ^*−/−*^MEFs (Fig. 5c), indicating the overall ability of MDM4 to control cell size. Importantly, the MDM4-dependent increase of cell size (FSC-H) is completely abolished upon RAPA treatment (Fig. 5d), indicating that these effects are mediated by the impairment of mTORC1 activity. Additionally, silencing of MDM4 significantly increased cell viability and cell number, whereas it was ineffective in the presence of RAPA (Fig. 5e and f, Additional file 1: Fig. S3b). Of note, the interference of S6K1 abrogated the effects of MDM4 on cell viability, confirming that MDM4 activity is mediated by this kinase (Fig. 5f). Overall, these data indicate that MDM4 controls mTORC1 activities towards cell size and growth.

**Fig 6:**
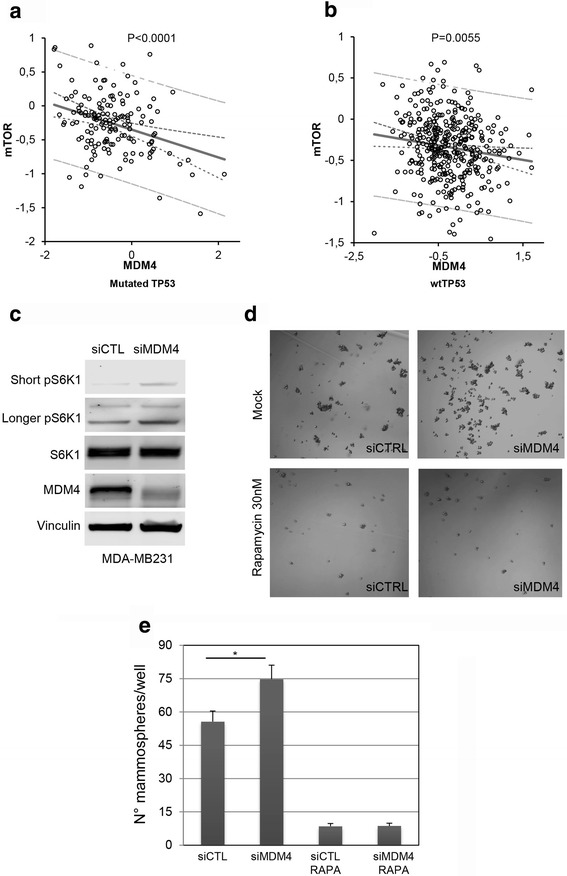
MDM4 regulates cell tumor cell growth . **a, b** Linear regression analysis between mTOR and MDM4 mRNA expression levels in Atlas breast cancer primary tumors characterized by mutated p53 (*N* = 151, *R* ^2^ = 0,11) or wild type p53 (wt TP53) (*N* = 375, *R* ^2^ = 0,02). **c** Wb analysis of the indicated proteins in MDA-MB-231 cells transfected with siMDM4 or siCTL and collected after 48 h. **d** Representative pictures of mammosphere formation in MDA-MB-231 treated as indicated in (c). **e** Quantification of mammosphere treated as in (d). Mean ± SD of three independent biological replicates is shown (* = *p* < 0.05 two‐tailed unpaired t‐test)

Mammalian TORC1 activities are instrumental to tumor growth. Consistently, many human tumors are characterized by increased activity and/or levels of mTOR signalling [13, 33, 34]. Given the inhibitory function of MDM4 towards mTORC1, we investigated a possible relationship between MDM4 and mTOR levels in human tumors by interrogating the Atlas database [35]. MDM4 expression has been associated with low risk/good prognosis in breast cancers independently of p53 [36] and high mTOR mRNA expression has been reported in breast tumors as well [37]. For this reason, we analysed MDM4 and mTOR in the database of human breast cancer. Interestingly, regression analysis showed a significant inverse correlation between *mTOR* and *MDM4* mRNA levels (Fig. 6a and b). This is highly significant in the tumors lacking wild-type p53 (Fig. 6a, *r* ^2^ = 0.11) suggesting the possible development of MDM4 anti-tumor activities especially in the absence of functioning p53. As control, no correlation was observed in normal breast tissues (data not shown). To support these data and ascertain the anti-oncogenic properties of MDM4, mammosphere forming assay was performed in breast tumor cell line MDA-MB-231, carrying R280K mutant p53 [38]. MDM4-KD caused increased pS6K1 levels in this cell line too (Fig. 6c). Interestingly, cells depleted for MDM4 showed an increased ability to form mammospheres compared to control cells (Fig. 6d and e) thus confirming the anti-oncogenic properties of MDM4. Rapamycin strongly reduced mammosphere formation indicating the sensitivity of these cells to mTORC1 inhibition and most importantly, abolished the effects of MDM4 depletion (Fig. 6d and e), supporting the functional link between mTOR and MDM4 in the maintenance of mTOR oncogenic properties.

## Discussion

The data presented in this work highlight a p53-independent link between MDM4 and mTOR, with MDM4 acting as inhibitor of mTORC1 kinase activity.

MDM4 acts at two different levels: 1) by anchoring the cytoplasmic inactive form of mTORC1, 2) by inhibiting the kinase function of the mTORC1. The activity of mTORC1 is regulated by different pathways that alter the composition and/or the post-translational modifications of the complex. Overall, these pathways affect the localization of mTORC1 that sways between its lysosomal active and its cytoplasmic inactive site [39]. This movement is accompanied by its association with activating complexes, represented by an activated heterodimer of Rag GTPases in presence of amino acids [31, 40, 41]. Most of the studies have therefore investigated how the cell senses the amino acids and activates mTORC1 [25]. Conversely, it is not known whether an active mechanism maintains mTOR in its inhibited state. By demonstrating that the lack of MDM4 increases the presence of mTORC1 at the lysosomes and the phosphorylation of its target p70S6K1, our data provide the proof of concept of an active mechanism able to control cytoplasmic inactive mTORC1. Furthermore, the ability of MDM4 to inhibit the kinase function of mTORC1 in vitro indicates the existence of an active inhibition of the mTOR enzymatic activity.

The binding of MDM4 to mTOR is stimulated by nutrient depletion suggesting a mechanism whereby MDM4 senses this condition. However, manipulation of MDM4 both in vitro and in vivo is able to alter mTORC1 localization and/or activity in normal growth conditions too, suggesting that the intracellular balance between the two proteins is determinant for the control of the growth-promoting function of mTORC1. The inverse correlation between MDM4 and mTOR observed in human breast cancer specimens is in agreement with this hypothesis.

Interestingly, the same MDM4 region is involved in p53 and mTOR binding, i.e. the N-terminus. Although no data have been reported about competitive activities between these two hubs of cell growth, the reduced correlation between MDM4 and mTOR observed in tumor harbouring wt-p53 compared to those with mutant p53 might indeed suggest an exclusive mode of MDM4 function. Furthermore, these data well reconcile with the reported anti-oncogenic properties of Mdm4 in absence of p53 [11].

The metabolism of tumor cells is emerging as an important field in which distinct metabolic pathways provide tumor cells of advantageous activities for their growth. In these last years, many studies have reported the frequent crosstalk between the pathways that control tumor development and cellular metabolism; accordingly, different oncogenes have demonstrated their ability to enhance and/or promote alternative ways of obtaining necessary nutrients thus establishing the hallmarks of cancer metabolism [42].

The data presented in this work add another member to this community, MDM4, endowed of p53-independent growth suppressive properties. This MDM4 function is in agreement with its pro-apoptotic activity under DNA damage and support a model of MDM4 with anti-oncogenic activities in stress conditions [43]. Furthermore, these data together with the previous report of MDM4 functioning as a bridge for phosphorylation of p53 [7], contribute to define MDM4 as a cytoplasmic scaffold ready to sense different stimuli – i.e. DNA damage, cell starvation – and to accordingly regulate cell growth by recruiting different partners.

## Conclusions

Overall, these data demonstrate a new p53-independent function of MDM4 in inhibiting mTOR. They highlight an additional way of de-regulation of mTORC1 activity in human tumors and include MDM4 among the proteins affecting both cell metabolism and tumorigenesis.

## Additional file

Additional file 1:
**Figure S1.** MDM4 inhibits S6K1 phosphorylation. **Figure S2.** MDM4 depletion leads to mTORC1 relocalization. **Figure S3** MDM4 affects cell size. (PDF 428 kb)

## Abbreviations

EBSS: Earle’s Balanced Salt Solution
eIF4: Eukaryotic initiation factore 4E-binding protein 1
HIPK2: Homeodomain Interacting Protein Kinase 2
MDM2: Mouse double minute 2 homolog
MDM4: Mouse double minute 4, human homolog of p53-binding protein
MEF: Mouse embryo fibroblast
mTORC1: Mammalian target of rapamycin complex 1
S6K1: Ribosomal protein S6 kinase beta-1
TOR: Target of rapamycin
