# Supplementary material for: MDM4 actively restrains cytoplasmic mTORC1 by sensing nutrient availability
Source: Mol Cancer. 2017 Mar 7;16:55. doi: 10.1186/s12943-017-0626-7 (PMC5341177; doi:10.1186/s12943-017-0626-7)
Supplement: Additional file 1: — Figure S1. MDM4 inhibits S6K1 phosphorylation. Figure S2. MDM4 depletion leads to mTORC1 relocalization. Figure S3 MDM4 affects cell size. (PDF 428 kb) [file 12943_2017_626_MOESM1_ESM.pdf]

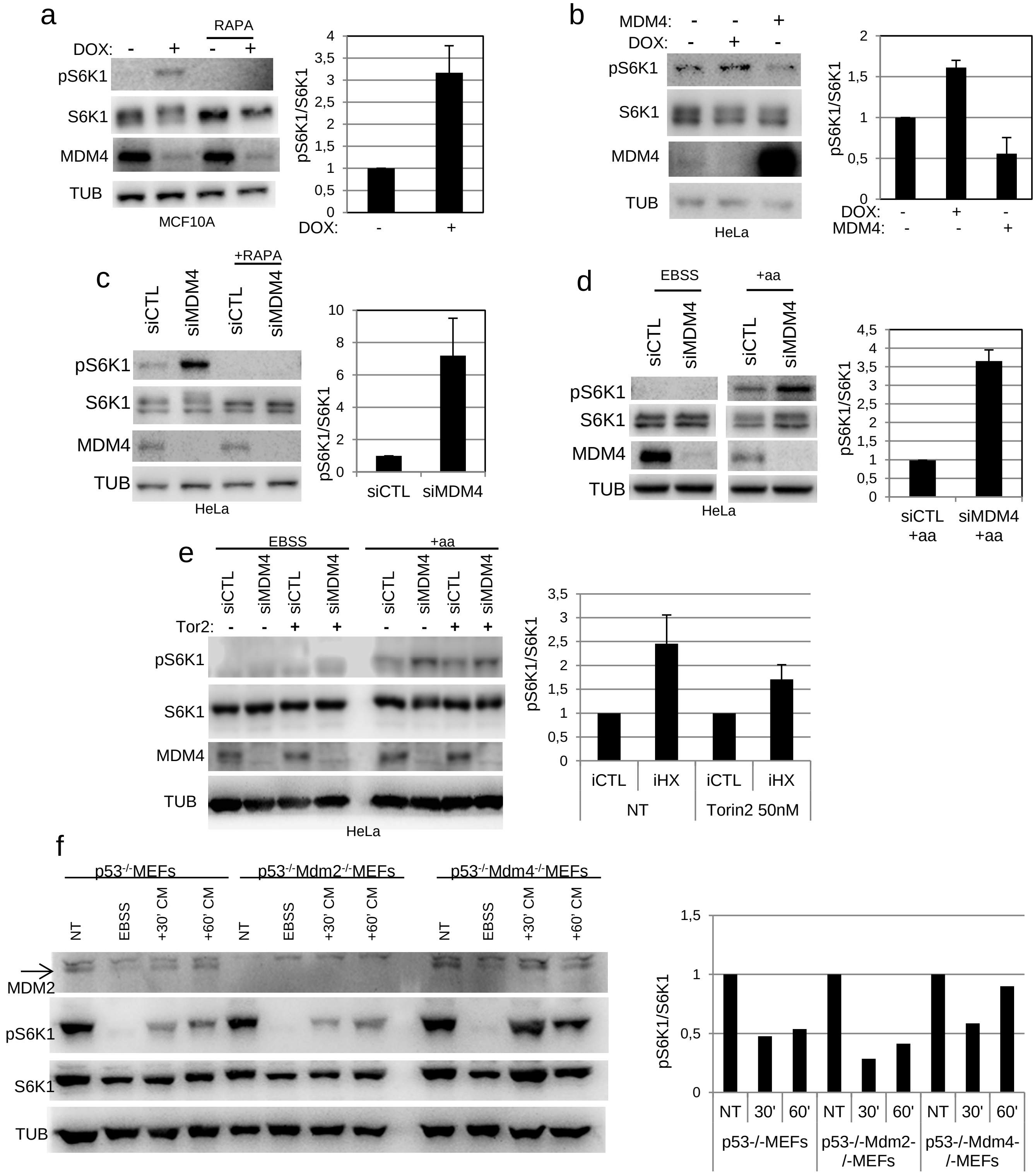

Figure S1, MDM4 inhibits S6K phosphorylation.

a Wb analysis of the indicated proteins in MCF10A cells interfered for MDM4 by inducible shRNA vector (by doxycycline-DOX) and/or transiently overexpressing MDM4. **b** Wb analysis of the indicated proteins in HeLa cells transfected with siCTL and siMDM4 and treated or not with RAPA. **c** Wb analysis of the indicated proteins in Hela cells transiently transfected as indicated and treated or not with RAPA (40nM). **d** Wb analysis of the indicated proteins in Hela cells transiently transfected as indicated and treated with EBSS for 50 minutes and then with aa for 30 minutes. **e** Wb analysis of the indicated proteins in Hela cells transfected with siMDM4 or siCTL and after 24hrs treated with Torin2 (Tor2, 50nm) for additional 24hrs. **f** Wb analysis of the indicated proteins in $p53^{-/-}$MEFs , $p53^{-/-}Mdm2^{-/-}$MEFs, $p53^{-/-}Mdm4^{-/-}$MEFs untreated (NT) or treated with EBSS for 50' and then with complete medium for additional 30' and 60'. Histograms show the ratio of densitometric values of phosphorylated S6K1 (pS6K1) to S6K1. pS6K1/S6K1 ratio from CTL lane was arbitrarily set to 1. In (f) the value in untreated cells (NT) of each cell line was arbitrarily set to 1. The graphs show the mean±SD of three independent experiments.

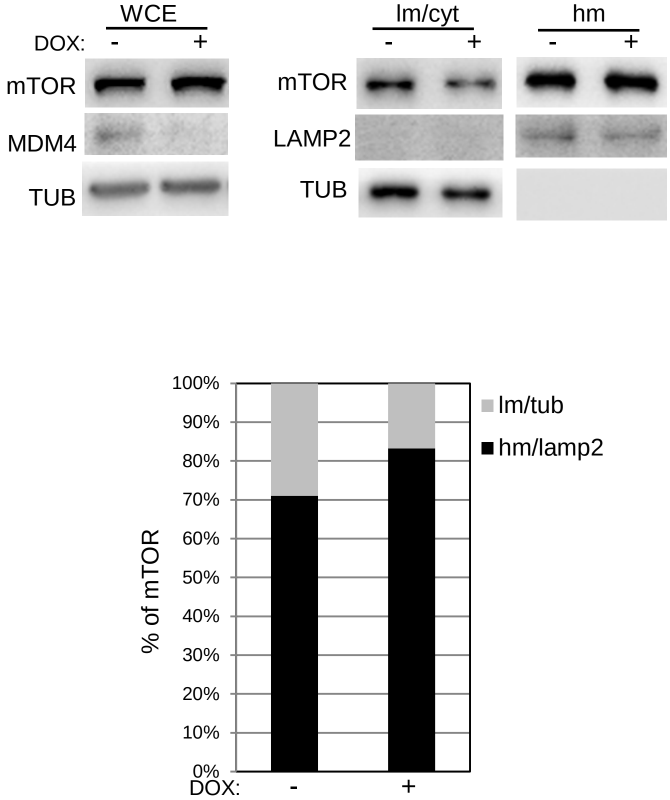

Figure S2, MDM4 depletion leads to mTORC1 relocalization.
Wb analysis of the indicated proteins in 293T cells interfered for MDM4 by inducible shRNA vector (by doxycycline-DOX). Left panel shows total whole cell extract (WCE), right panels show light membrane/cytosolic (lm/cyt) and heavy membrane (hm) fractions. Histogram shows the % of mTOR signal in the lm/cy and hm fractions corrected for the respective loading control. mTOR signals in the lm+hm fractions was arbitrarily set to 100%.

a

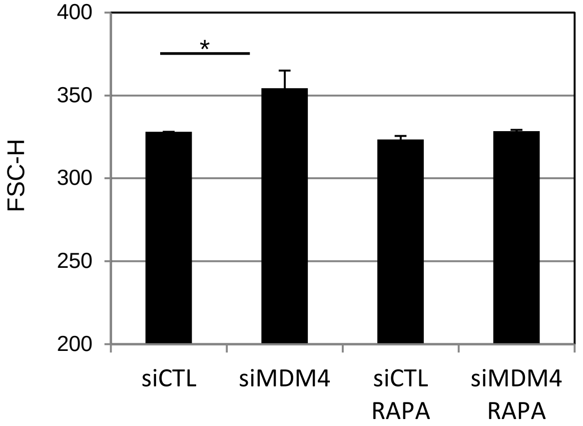

b

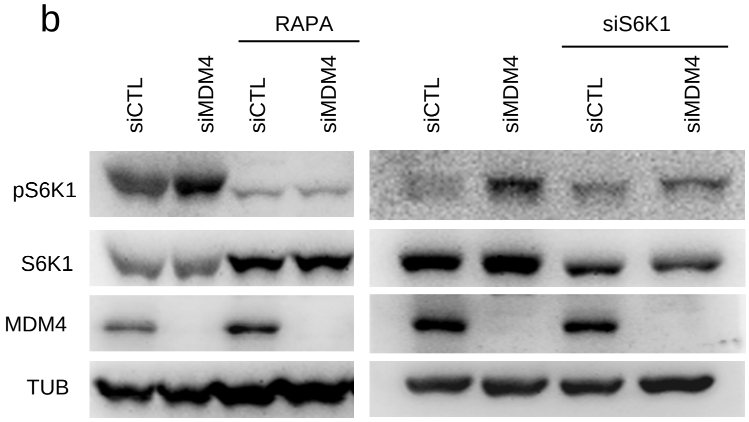

Figure S3, MDM4 affects cell size.

a FSC-H of Hela cells treated as indicated. Mean±SD of two independent biological replicates performed in duplicates is shown. **b** Wb analysis of the indicated proteins in 293T cells transfected with siCTL or siMDM4 and after 24hrs treated with RAPA or transfected with siS6K1 for additional 24hrs.
